# Infant feeding practices in the Saharawi refugee camps Algeria, a cross-sectional study among children from birth to six months of age

**DOI:** 10.1186/s13006-016-0098-1

**Published:** 2017-01-28

**Authors:** Inger Aakre, Anne Marie Lilleengen, Marie Lerseth Aarsand, Tor A. Strand, Ingrid Barikmo, Sigrun Henjum

**Affiliations:** 1 Department of Nursing and Health Promotion, Faculty of Health Sciences, Oslo and Akershus University College, PB 4 St. Olavs plass, 0130 Oslo, Norway; 2 Department of Global Public Health and Primary Care, Faculty of Medicine and Dentistry, University of Bergen, PB 7804, N-5018 Bergen, Norway; 3 Medical Microbiology, Department of Laboratory Medicine, Medical Services Division, Innlandet Hospital Trust, Anders Sandvigs gate 17, 2609 Lillehammer, Norway

**Keywords:** Breastfeeding, Infants, Undernutrition, Refugees, Infant feeding practices

## Abstract

**Background:**

Appropriate breastfeeding and infant feeding practices are crucial to a child’s growth and development. The objective of this paper is to describe breastfeeding and general feeding practices and the nutrition status among children from birth to 6 months of age, in the Saharawi refugee camps located in Algeria.

**Methods:**

A cross-sectional study was carried out among 111 lactating mothers with infants from birth to 6 months of age. Data regarding breastfeeding practices and a 24 h dietary recall for the infants were collected to assess the World Health Organization’s (WHO) indicators for infant and young child feeding. For exclusive and predominant breastfeeding, age disaggregation for each month was applied to the data. Background characteristics from the mothers and infants were collected, together with anthropometrical measures. We explored predictors for breastfeeding and nutrition status in multiple regression models.

**Results:**

In total 13.8%, 8.2% and 16.5% of the infants were stunted, wasted and underweight, respectively. Approximately 65% initiated breastfeeding within 1 h after birth and 11.7 and 21.6% were exclusively or predominantly breastfed less than 6 months. The most commonly given solid foods were dates (27.0%) and bread (10.8%). In multiple regression models, initiation of breastfeeding within 1 h after birth gave increased probability of exclusive or predominant breastfeeding. Giving birth at home as opposed to in a hospital and increasing number of children gave increased probability of initiating breastfeeding early. Exclusive or predominant breastfeeding seemed to protect against underweight and wasting.

**Conclusions:**

Exclusively or predominant breastfeeding was low among Saharawi refugee infants. Wasting and underweight was common and more likely to occur if the infants were not exclusively or predominantly breastfed. These findings support the current international breastfeeding recommendations, and suggest that there is an urgent need for promoting infant feeding practices in the Sahara refugee camps.

## Background

Optimal infant and young child feeding is crucial for health and development [1]. Women are recommended to breastfeed exclusively for 6 months and to continue breastfeeding until the child is 2 years [2]. Breastfeeding is widely acknowledged as the best and safest form of infant feeding; containing most of the necessary nutrients and bio-active components needed the first 6 months of life [3] and it further protects the infant from acute infectious illnesses, such as gastroenteritis, respiratory disease and other infections [4, 5]. To emphasize the importance of breastfeeding, the recent published Lancet Breastfeeding series [6], have estimated that scaling up of breastfeeding could prevent 823,000 child deaths every year. Breastfeeding has not only been found to be important in early childhood, a systematic review of the long-term effects of breastfeeding found that breastfeeding may protect against high blood pressure, diabetes, overweight and obesity in adult life, and further improve cognitive development [7]. Breastfeeding has also been found to be beneficial for mothers, and may prevent breast cancer and improve birth spacing [8]. Despite all the well documented benefits of breastfeeding, promotion and support for breastfeeding is still needed in many communities. As pointed out by Rollins et al., breastfeeding needs supportive measures at different levels, from political and legal, to social attitudes and health-care services. Scaling up for breastfeeding would not only prevent child mortality and morbidity, but also improve food security, education, equity, development and environmental issues [9].

WHO has developed a set of indicators to assess feeding practices among infants and young children [2]. The two core indicators for children less than 6 months of age are exclusive breastfeeding and early initiation to breastfeeding. Exclusive breastfeeding the first 6 months of life has been associated with reduced risk of infectious diseases and reduced mortality rates [4, 10]. Early initiation to breastfeeding, also referred to as timely initiation, is further recommended as it increases the likelihood of exclusive breastfeeding and reduced child mortality [10]. Further, it ensures that the newborn receives the colostrum with its beneficial effects [11]. Recent data show that breastfeeding practices in low- and middle-income countries are poor, where only 37% is exclusively breastfed less than six months, and about 40% is early initiated to breastfeeding [6].

The Global Nutrition Report 2016, states that even if the nutrition situation has improved dramatically over the past decades, the current global prevalence of malnutrition is still high, with stunting and wasting prevalence of 23.8 and 7.5%, respectively [12]. In emergencies, either acute or long-term, infants or young children are a particularly vulnerable group. In refugee populations, the highest mortality rates are seen among children aged less than 5 years of age [13], and for children less than 1 year published total mortality rates range from 12% to 53% [14–16]. Diarrhea and respiratory tract infections, both associated with malnutrition, are the most common causes of death among children in emergencies [13]. In refugee situations, the risk of malnutrition, infectious diseases and death are dramatically heightened in children less than 2 years of age who are not breastfed, and in children less than 6 months of age who are not exclusively breastfed [17].

Refugees from Western Sahara have been living in refugee camps in the Algerian desert since 1975, located near the city of Tindouf. Western Sahara was previously a Spanish colony, and when Spain withdrew in 1975 Morocco occupied the country. A large part of the Saharawi population fled to the Algerian desert, where refugee camps were established to accommodate them. The approximately 165 000 refugees live in a harsh desert environment, which makes it difficult to cultivate food crops and with limited water supply. The refugee population is totally dependent upon food aid, which are mainly provided by the World Food Program (WFP), United Nations High Commissioner for Refugees (UNHCR) and different other non-governmental organizations [18]. Access to additional foods is limited, but there are small shops which offer meat, milk and other fresh foods. However, few of the refugees possess the means to buy any quantities of these foods.

A previous study among the Saharawi refugees showed that the prevalence of undernutrition was high among children 6–59 months of age, with a wasting, stunting and underweight prevalence of 9.1%, 29.1% and 18.6% respectively [19]. A report from a UNHCR nutrition survey from 2010 showed that breastfeeding practices were inadequate [20]; only 18.4% of the children less than 6 months were exclusively breastfed. In our study, we have collected more thorough data regarding the infant feeding situation and their nutritional status. In this paper, we describe breastfeeding and infant feeding practices, and the nutritional status among Saharawi children from birth to 6 months of age; in addition we explore possible predictors for breastfeeding practices and nutrition status. To our knowledge, this is the first scientific paper that describes infant feeding practices among the Saharawi refugees. This paper may therefore be an important contribution in order to document the Saharawi refugee’s situation in the scientific literature.

## Methods

A cross-sectional study was conducted from October to December 2010, in the Saharawi refugee camps. Data regarding breastfeeding and infant feeding practices were collected as a part of a larger health facility based nutrition study [21]. The target population was breastfeeding women with children 0–6 months of age, which were included by convenience sampling [22]. A total of 111 mother-infant pairs participated in the study and a detailed description on sample calculation and selection procedure has previously been described [21].

The participants answered a pre-coded questionnaire concerning background variables, breastfeeding and general feeding practices. A 24 h dietary recall without quantity measures were collected from the mothers on behalf of their children. The questionnaire and 24 h dietary recall were administered by interview in the local language Hassaniya, by trained fieldworkers and supervised by the field supervisor.

The WHO’s indicators for assessing infant and young child feeding [2] were used for the indicators accessible in our material, which were; exclusive breastfeeding, predominantly breastfeeding and early/timely initiation to breastfeeding. Exclusive breastfeeding requires that the child has been given only breast milk, no food, liquids or formula is allowed with the exception of medicines, vitamin and mineral supplements. Predominant and breastfeeding requires the child to be breastfed as the main source of nourishment, and allows in addition certain liquids such as water, water based drinks, fruit juice and ritual fluids (i.e. tea). For exclusive or predominant breastfeeding, age disaggregation for each month was applied in the data management. Early/timely initiation of breastfeeding requires the child to be put to the breast within 1 h after birth. The concept of completed age [23] has been used when presenting indicators within in age groups. For exclusive or predominate breastfeeding the ages 0–6 months have been used, instead of 0–5 months which are used in the WHO indicators.

In addition to the WHO indicators for infant and young child feeding, different categories for infant feeding practices is presented in the results section. These categories are; “breast milk and water only”, “breast milk and other milk/formula only”, “given liquids” and “given complementary foods”. All the categories were made based on the 24 h recall. The category “breast milk and water only” included plain water and in addition, oil water and sugar water which is common among the refugees. The category “breast milk and other milk/formula only” included infant formula, goat milk, camel milk and cow’s milk. The category “given liquids” included all drinks given such as milk, water and fruit juices. The category “given complementary foods” contains all complementary solid and semisolid or soft foods given, such as bread, rice, porridge, yogurt, fruit and vegetables etc., but not liquids.

Body weight was measured using a UNICEF digital platform scale (SECA 890, Hamburg, Germany). The infants’ weight was measured when being held by its mother after resetting the weight scale. The height and length were measured to the nearest 0.1 cm using a portable UNICEF length board. Children’s nutrition status was determined using the WHO gender and age specific *z*-scores for the indicators: height-for age (HAZ), weight-for-age (WAZ) and weight-for height (WHZ) [24]. Z-scores were calculated using the WHO macro for SPSS [25]. The measures HAZ, WAZ and WHZ, also referred to as stunting, underweight and wasting, were used to assess undernutrition when the z-score was below −2 standard deviations from the median of the reference population [26].

### Data management and statistics

Data were analyzed using IBM SPSS version 22 (IBM Corp. Armonk, NY). Normally distributed data was presented as means and standard deviations (SD), skewed data was presented as medians and 25 to 75 percentiles (p25-p75). In logistic regression analysis, exclusive or predominant breastfeeding was merged into one variable due to low prevalence of exclusively breastfeeding, and used as a binary variable, where 0 = not exclusive or predominant breastfeeding and 1 = exclusive or predominant breastfeeding. Exclusive or predominant breastfeeding was tested for associations with the following independent variables in crude models; BMI mother, age mother, age child, education mother, work mother, gender child, prelacteal feeds, early/timely initiation to breastfeeding, number of children, birthplace, diarrhea last 2 weeks and other disease last 2 weeks. Early/timely initiation of breastfeeding was assessed for associations with the following independent variables in crude models; BMI of mother, age of mother, education of mother, work of mother, gender of child, number of children and birthplace. Categorical variables with more than two categories were dichotomized using following categories; for education mother 0 = 6^th^ grade or less, 1 = more that 6^th^ grade, for birthplace 0 = at home, or local health station, 1 = hospital. All explanatory variables showing an association (*p* < 0.05) were included in multiple models, and only variables still significant were retained in the final models.

To assess whether breastfeeding practises had influence of the children’s nutrition status, we also included HAZ, WAZ and WHZ as dependent variables in simple and multiple linear regression models, to assess breastfeeding practises' influence on attained growth. In the adjusted model exclusive or predominant breastfeeding was adjusted for child’s age, disease and diarrhea last 2 weeks and mothers BMI. All regression models were checked for homoscedasticity using standard residuals within ± 3 and cook’s distance < 1 as parameters.

### Ethical considerations

Ethical approval for the study was given by the Regional Committees for Medical and Health Research Ethics in Norway (Reference 2010/2513), and by the Saharawi Ministry of Public Health. Study information was provided both orally and written to the participants. Written informed consent was given by the mothers on behalf of themselves and their children.

## Results

Table 1 shows the background characteristics of the women in the study. The mean ± SD age of the women was 31.4 ± 5.9 years and the mean weight and height were 65.9 kg, and 156.5 cm respectively. The mean ± SD BMI was 26.9 ± 4.6, and 67.5% of the women were overweight or obese. There were 22.7% of the women with education ≥ 10^th^ grade, and 18.9% reported that they currently were working outside the home. The mean household size consisted of 5.1 people, and the women had in average 3.1 living children.

**Table 1:** Background characteristics of Saharawi women (*n* = 111)^a^

Women (*n* = 111)	
Age, years	31.4 ± 5.9
Height, cm	156.5 ± 5.4
Weight, kg	65.9 ± 11.7
BMI kg/m^2^	26.9 ± 4.6
<18.5	2.7 [3]
18.5–24.9	29.7 [33]
25.0–29.9	43.2 [48]
≥30	24.3 [27]
Education*, years	
≤6	32.7 [36]
7–9	44.5 [49]
≥10	22.7 [25]
Currently working	18.9 [21]
Household size, *n*	5.1 ± 1.9
Number of children, *n*	3.1 ± 1.9
Health station attendance	91.1 [100]

^a^Values are presented as mean ± SD, median (IQR), and % [*n*]. * One missing from education

In Table 2, background characteristics and nutrition status among the children are presented. The mean ± SD age of the children was 3.0 ± 1.8 months and 63.6% children were female. Approximately 52% were born at home, 5% at the local health station and 43% were born at a hospital. One third of the children have had diarrhea and/or other disease the last 2 weeks. Data regarding nutritional status showed that totally 8.2% of the children were wasted, 16.5% underweight and 13.8% stunted, of which 0.9% were severely wasted, 6.4% severely underweight and 3.7% were severely stunted.

**Table 2:** Background characteristics and nutrition status among Saharawi children (*n* = 111)^a^

Children (*n* = 111)	
Age, months	3.0 ± 1.8
Male	36.4 [40]
Female	63.6 [70]
Diarrhea last 2 weeks	32.4 [36]
Other disease last 2 weeks	35.1 [39]
Birthplace	
Home	52.3 [58]
Health station	4.5 [5]
Hospital in camp	24.3 [27]
Central hospital	18.9 [21]
Nutritional status	
Wasting*	−0.3 ± 1.2
Moderate, −3 and ≤ −2 WHZ	7.3 [8]
Severe, ˂−3 WHZ	0.9 [1]
Total, ≤−2 WHZ	8.2 [9]
Underweight*	−0.8 ± 1.3
Moderate, −3 and ≤− 2 WAZ	10.1 [11]
Severe, ≤-3 WAZ	6.4 [7]
Total, ≤−2 WAZ	16.5 [18]
Stunting*	−0.6 ± 1.2
Moderate, −3 and ≤ −2 HAZ	10.1 [11]
Severe, ≤−3 HAZ	3.7 [4]
Total, ≤-2 HAZ	13.8 [15]

^a^Values are presented as mean ± SD, median (IQR), and % [*n*].* Two children missing from stunting, wasting and underweight (*n* = 109)

Breastfeeding and feeding practices are described in Table 3. There were 64.9% of the children who were put to the breast within 1 h after birth, 14.4% within the first six hours and 20.7% of the children initiated breastfeeding after more than 6 h. As many as 23.4% of infants were given water or formula before initiating breastfeeding. In total 11.7% and 21.6% were exclusively or predominantly breastfed, respectively. In addition to breastmilk there were 70.3% of the children who was given water as well, and 42.3% were given other milk or formula. In total, 81.1% had received some sort of liquids, such as milk, juice, water in addition to breastmilk. As many as 43.2% of the children had received complementary foods, not including drinks, in addition to breastmilk.

**Table 3:** Breastfeeding practises among Saharawi children (*n* = 111)^a^

Breastfeeding practices	
Initiation of breastfeeding	
Within 1 h of birth	64.9 [72]
Within the first 6 h	14.4 [16]
Within the first day	6.3 [7]
Within the second day	5.4 (6)
Within the third day	4.5 [5]
More than 3 days	4.5 [5]
Prelacteal feeds	23.4 [26]
Sugar water	22.5 [25]
Oil water	12.6 [14]
Infant formula	0.9 [1]
Saline solution	0.9 [1]
Breastfeeding frequency day	
1–5 times	28.8 [32]
6–10 times	11.7 [13]
11–15 times	1.8 [2]
>15 times	57.7 [64]
Breastfeeding frequency night	
No times	0.9 [1]
1–5 times	57.7 [64]
6–10 times	7.2 [8]
11–15 times	0.9 [1]
>15 times	33.3 [37]
Breastfeeding on demand	86.5 [96]
Exclusively breastfed	11.7 [13]
<3 months	16.0 [8]
>3 months	8.5 [5]
Predominantly breastfed	21.6 [24]
Breast milk and water only	70.3 [78]
Breast milk and other milk/formula only	42.3 [48]
Given liquids	81.1 [90]
Given complementary foods^b^	43.2 [48]

^a^Values are presented as mean ± SD, median (IQR), and % [*n*]. ^b^ Includes solid, semi solid and soft foods, but no liquids

**Fig. 1:**
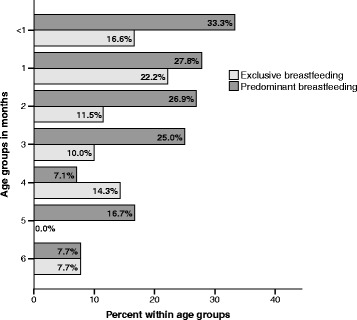
Prevalence of exclusively and predominantly breastfeeding among Saharawi children aged 0–6 months within age groups (total *n* = 109, for age groups; < 1 months *n* = 6, 1 months *n* = 18, 2 months *n* = 26, 3 months *n* = 20, 4 months *n* = 14, 5 months *n* = 12 and 6 months *n* = 13)

In Fig. 1, age disaggregation for exclusive or predominant breastfeeding is shown. Both exclusive and predominant breastfeeding decreases as the children’s age increases. At age, < 1 month 16.7% and 33.3% were exclusively or predominantly breastfed respectively, while at 6 months age 7.7% were both exclusively or predominantly breastfed.

**Fig. 2:**
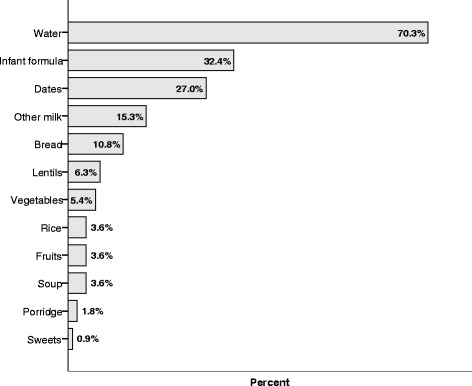
Types of foods and drinks given last 24-h among Saharawi children aged 0–6 months (*n* = 111)

Figure 2 illustrates the different types of foods and liquids given in the last 24 h. Water was most frequently given; 70.3% had been given water the previous day, 32.4% had been given infant formula and 15.3% had received other milk, which were mostly camel and/or goat milk. The most frequent given solid food was dates, and 27.0% of infants were given dates the previous day. There were also 10.8% that had been given bread, 6.3% had received lentils and 5.4% had received vegetables where carrots and potatoes were the most common. The other foods given were soup, porridge, fruit, sweets and rice.

Table 4 show associations between the breastfeeding indicators exclusively or predominantly breastfeeding, and early initiation of breastfeeding. Exclusively or predominantly, breastfeeding was significantly associated with age and early initiation to breastfeeding in the multivariate model. Increased child age gave decreased probability of exclusive or predominant breastfeeding with an adjusted OR (95% CI) of 0.70 (0.53, 0.92). For early initiation to breastfeeding the adjusted OR (95% CI) was 3.20 (1.20, 8.54), meaning that early initiation to breastfeeding gives increased probability of exclusive or predominant breastfeeding. In the multivariate model for early initiation of breastfeeding, we found a significant association with birthplace and number of children in the family. For birthplace, there was an decreased probability of early initiation of breastfeeding if the child was born at a hospital as opposed to at home or at the local health station, with an adjusted OR (95% CI) of 0.42 (0.18, 0.99). Further there were increased probability of initiating breastfeeding early as number of children in the family increased with an adjusted OR (95% CI) of 1.41 (1.07, 1.87).

**Table 4:** Predictors for exclusively or predominantly breastfeeding and early initiation of breastfeeding among Saharawi children (*n* 111)

Dependent variables^a^	Predictor Variables	Unadjusted OR (95% CI)		Adjusted OR (95% CI)	
			*p*		*p*
Exclusivley or	Age *(months)*	0.71 (0.55, 0.93)	*0.011*	0.70 (0.53, 0.92)	*0.011*
predominantley breastfeeding	Early initiation^b^	3.27 (1.27, 8.38)	*0.014*	3.20 (1.20, 8.54)	*0.020*
Early initiation of breastfeeding	Birthplace^c^	0.31 (0.14, 0.70)	*0.005*	0.42 (0.18, 0.99)	*0.047*
	Number of children *(n)*	1.52 (1.16, 2.00)	*0.003*	1.41 (1.07, 1.87)	*0.015*

^a^ *n=*109 for the dependent variable exclusively and/or predominantly breastfeeding. Tested with logistic regression. ^b^ Categories for early initiation to breastfeeding: 0 = no, 1 = yes. ^c^ Categories for birthplace: 0 = at home/health station, 1 = hospital

The influence of exclusive or predominant breastfeeding on nutrition indices HAZ, WAZ and WHZ is presented in Table 5. The mean z-score was significantly higher for WAZ and WHZ among the children who were exclusively or predominately breastfed as opposed to those who were not, showing a mean difference and 95% CI of 0.65 (0.14, 1.15) and 0.52 (0.004, 1.01) respectively. Breastfeeding practices were not associated with HAZ. After adjusting for child’s age, disease and diarrhea and mothers BMI, WAZ was still significantly higher among the children who had been exclusively or predominantly breastfed with an adjusted mean difference and 95% CI of 0.62 (0.010, 1.13). While for WHZ it was no longer a significant association with exclusive or predominant breastfeeding showing a mean difference and 95% CI of 0.41 (−0.01, 0.91), and disease seemed to be the main influential variable.

**Table 5:** Exclusive and predominant breastfeeding’s unadjusted and adjusted changes on attained growth among Saharawi children

	Not predominant or exclusively breastfed (*n* =73)	Predominant or exclusively breastfed (*n* = 35)	Mean difference (95% CI)	Adjusted mean difference (95% CI)
HAZ	−0.71 (1.2)	−0.56 (1.2)	0.12 (−0.34, 0.60)	0.18 (−0.31, 0.66)
WAZ	−0.96 (1.3)	−0.31 (1.2)	0.65 (0.14, 1.15)*	0.62 (0.10,1.13)*
WHZ	−0.52 (1.3)	0.01 (1.07)	0.52 (0.04, 1.01)*	0.41 (−0.08, 0.91)

Data are presented as mean (SD) unless otherwise indicated. Adjusted mean difference was adjusted for child’s age, disease and diarrhea last 2 weeks and mothers BMI, in linear multiple regression models. One outlier removed from HAZ (*n* = 108), Two outliers removed from WAZ (*n* = 107). No outliers removed from WAZ (*n* = 109). * *p* < 0.05

## Discussion

We found that breastfeeding practices was poor among the Saharawi refugee children; only 11.7% and 21.6% were exclusively or predominantly breastfed, respectively. Further, as many as 43.2% were given solid, semi solid or soft foods despite their young age. There were 64.9% which were early initiated to breastfeeding. In multiple regression models, exclusive or predominant breastfeeding seemed to protect against underweight.

### Breastfeeding practices

Initiation of breastfeeding within 1 h after birth has numerous nutritional and immunological benefits and has been found to reduce neonatal mortality [11, 27]. A number of potential mechanisms for the observed reduction in mortality have been suggested and include the early stimulation of the immune system through exposure to the high levels of immunoglobulins and lymphocytes found in colostrum, along with the displacement of pre-lacteal feeds which may increase infections and also disrupt normal gut maturation [11, 28]. Despite these recognized benefits of early initiation of breastfeeding, only about 40% of the world’s newborns are put to the breast within 1 h after birth [29]. The rates in Africa are generally low, and the World Nutrition Report from 2015 revealed that 50% initiated breastfeeding early on the African continent, compared to 39% in the West African region [12]. In a study by Engebretsen et al., early initiation of breastfeeding was ranging from 3.6 to 13.4% in low income countries such as Burkina Faso and Uganda up to 47.1% in middle income countries such as South Africa [30]. The proportion of women who initiated breastfeeding early in our study was 64.9%, which compared to the study mentioned above and the international rates of 40% may be considered quite high, although it is far from optimal.

We found an association between early initiation of breastfeeding and birthplace, meaning that children born at home more frequently were early initiated to breastfeeding than children born in a hospital. There is little evidence to support this association in the literature. However, the norm among women in the Saharawi culture is to give birth at home. Women who give birth in hospital do it because of being at risk for complications. It is known that early initiation of breastfeeding may be delayed after complications such as caesarian section [31]. We also found an association between early initiation of breastfeeding and the number of children the mother had. The reason why increased number of children is associated with early initiation to breastfeeding in our study is unclear. In a global breastfeeding survey, data from two African sites showed similar results as ours; nulliparity seemed to give increased risk of not initiate breastfeeding early [32]. The authors of this study suggest that this finding could be related to interplay between several factors such as maternal age, lack of knowledge and cultural beliefs. A study among Arab women in the Middle East showed that multiparous women were more likely to initiate breastfeeding early than mothers who had given birth once before, which also supports our finding [33]. Factors associated with early initiation of breastfeeding in other surveys were; giving birth at a health facility, larger birth weight and agricultural occupation, which were the case in a large national survey on breastfeeding among Nepalese mothers [34]. While in a Tanzanian survey [35], giving birth at a health facility and vaginal birth was the main predictors for early initiation, and that mother’s knowledge of newborn danger signs were negatively associated [35].

Prelacteal feeds was quite commonly given to the children, and sugar- and/or oil water was the most used. Despite its harmful effects, prelacteal feeds is commonly given in many low-income countries; studies from Egypt, Ethiopia and Kenya found that prelacteal feeds was given 57.8, 38.8 and 26.8% of the children [36–38]. The Saharawi people are Muslims, and in some Muslim communities, prelacteal feeds, such as sugar water, may be used the first day after birth because it is believed that the colostrum has low nutritional value and may be considered dirty [36, 39]. We do not know whether this was a factor influencing the refugee women in our study.

Even if the benefits of breastfeeding are innumerable [40], only 39% of all African infants less than 6 months of age are exclusively breastfed [12]. West Africa has one of the lowest rates in the world, with countries such as Guinea recording 21% and Côte d’Ivoire 12%, and a total prevalence of 26% [12]. In our study, the prevalence of exclusive breastfeeding less than 6 months was 11.7%, which corresponds with the low rates from the region. A study among 593 mothers from the United Arab Emirates found that 7.4% of the children were exclusively breastfed up to 4 months of age, while only 1.9% was exclusively breastfed until 5 months of age [33]. Exclusive or predominant breastfeeding had a negative association with child’s age and a positive association with number of children in the family, in the multivariate regression analyses. The reduction of exclusively or predominant breastfeeding by age is well documented [41]. Further there seemed to be a greater probability of exclusive or predominant breastfeeding among the children who had been initiated early to breastfeeding, which also is well documented [10]. Our data show that as many as 32.4% were given infant formula. It is widely known that promotion of infant formula reduces breastfeeding [42, 43]. However, we do not know the practice of formula marketing in the refugee camps, or if this could be a possible explanation for the low breastfeeding rates.

Despite the children’s young age, complementary solid, semi solid or soft foods were commonly given, and as many as 43.2% had received either solid, semi solid or soft foods, which included foods such as lentils, bread, rice and dates. In a study from Tanzania, exclusive breastfeeding was rare, and solid foods seemed to be introduced before the age of 6 months, and semi solid foods was introduced already from 1 month’s age among some of the children [44]. In a recent study among seven West African countries, the introduction of complementary foods was commonly seen among children 3–5 months of age, and were further associated with diarrhea and acute respiratory infections [45].

### Nutritional status

We found a high prevalence of wasting, stunting and underweight among the Sahrawi infants. According to WHO, a wasting prevalence of < 5% is acceptable, while prevalence between 5 and 9% is considered poor, which will apply for our sample having a wasting prevalence of 8.2%. The acceptable cut off prevalence for underweight is < 10%, while 10–19% is considered as medium significant for public health, according to WHO. In our data, the underweight prevalence was 16.5%, which exceeds the cut-off for an acceptable prevalence. We found 13.8% stunting, which is a low prevalence in relation to public health significance [46]. Even so, the stunting prevalence could be considered alarming when taking into account the children’s young age.

It is well documented that breastfeeding protects against undernutrition [6, 47]. In our study exclusive or predominant breastfeeding seemed to protect against underweight (WAZ) and wasting (WHZ) in the simple model, but after adjusting for disease, child’s age and mothers BMI, WAZ was no longer a significant associated with breastfeeding. Disease on the other hand seemed to have a large influence on the wasting prevalence than breastfeeding practices, which is expected [48]. Exclusive and predominantly breastfeeding did not seem to be associated with stunting (HAZ). It should be noted that stunting is a reflection of chronic malnutrition (that follows acute malnutrition) and is more common later in infancy. The consequences of suboptimal feeding practices on stunting will accordingly not appear within the first few months of life. In a recent study from Malawi, exclusive breastfeeding for 6 months was associated with increased length-for-age among children between 6 and 12 months, and a stronger association was seen among the older infants [49]. The study points out that the influence of the beneficial effects of breastfeeding increases as the child’s age increases, therefore in our study we might could have found an association between HAZ and breastfeeding status at a later point in time.

### Strengths and Limitations

An inclusion criterion in this study was that the mother should breastfeed. Accordingly, the prevalence of exclusive or predominant breastfeeding in this study is among breastfeeding women, and not among all women with children less than 6 months of age. In the recruitment process, 150 women were asked to participate; of which 17 were excluded because they were not breastfeeding, giving a breastfeeding prevalence of 88.7%. However, our data gives valuable information of infant feeding and breastfeeding practices among the women who do breastfeed, and despite the low prevalence of exclusive and predominant breastfeeding we know that these numbers probably are overestimating compared to all mothers with children less than 6 months of age. Further, the prevalence of exclusive breastfeeding would depend upon the methods used for data collection. In our study a 24 h recall was used, which may according to the WHO also overestimate the prevalence [2, 50]. There are also known weaknesses to the 24 h dietary recall method, in regard of misreporting foods [22]. However, a checklist was used in addition to the recall adapted from the WHO Infant and Young Child Feeding Module [23], to help the participants to remember. An unpublished report from a nutrition survey in the Saharawi refugee camps by UNHCR from 2011 [20], revealed the prevalence of exclusive breastfeeding among infants < 6 months of age to be 18.4%, and 94.5% who ever breastfed, which corresponds well with our results.

## Conclusions

Breastfeeding practices are poor among the Saharawi refugees, and we found an association between poor breastfeeding practices and undernourishment. Exclusive or predominant breastfeeding had increased probability if the mother had initiated breastfeeding early. Early initiation of breastfeeding had an increased probability if the child was born at home, and with multiparty. Promotion of optimal breastfeeding practices should be promoted in the refugee camps to achieve better infant feeding practices and prevent undernourishment among the children. Further, the mother’s current knowledge of infant child feeding should be examined, and whether promotion of exclusive breastfeeding and early initiation is a current practice in the refugee camps.

## Abbreviations

BMI: Body mass index
CI: Confidence interval
HAZ: Height-for-age z-score
IYCF: Infant and young child feeding
OR: Odds ratio
p25: 25^th^ percentile
p75: 75^th^ percentile
SD: Standard deviation
UNHCR: United Nations High Commissioner for Refugees
UNICEF: United Nations Children’s Fund
WAZ: Weight-for-age z-score
WHO: World Health Organization
WHZ: Weight-for-height z-score
